# Rapid Implementation of Telegenetic Counseling in the COVID-19 and Swedish Healthcare Context: A Feasibility Study

**DOI:** 10.3389/frhs.2022.848512

**Published:** 2022-06-23

**Authors:** Rebecka Pestoff, Peter Johansson, Henrik Danielsson, Margit Neher, Cecilia Gunnarsson

**Affiliations:** ^1^Centre for Rare Diseases in Southeast Region of Sweden, Linköping University, Linköping, Sweden; ^2^Division of Community Medicine, Department of Medical and Health Sciences, Linköping University, Linköping, Sweden; ^3^Department of Clinical Genetics, Biomedical and Clinical Sciences, Linköping University, Linköping, Sweden; ^4^Department of Health, Medicine and Caring Sciences, Linköping University, Linköping, Sweden; ^5^Department of Behavioural Sciences and Learning, Linköping University, Linköping, Sweden; ^6^Department of Rehabilitation, School of Health and Welfare, Jönköping University, Jönköping, Sweden; ^7^Department of Clinical and Experimental Science, Linköping University, Linköping, Sweden

**Keywords:** communication, genetic counseling, genetics services, service delivery models, telemedicine

## Abstract

This study reports the process and preliminary findings of rapid implementation of telegenetic counseling in the context of Swedish healthcare and COVID-19 pandemic, from both a patient and a provider perspective. Fourty-nine patients and 6 healthcare professionals were included in this feasibility study of telegenetic counseling in a regional Department of Clinical Genetics in Sweden. Telegenetic counseling is here defined as providing genetic counseling to patients by video (*n* = 30) or telephone (*n* = 19) appointments. Four specific feasibility aspects were considered: acceptability, demand, implementation, and preliminary efficacy. Several measures were used including the Genetic Counseling Outcome Scale 24 (collected pre- and post-counseling); the Telehealth Usability Questionnaire; a short study specific evaluation and Visiba Care evaluations, all collected post-counseling. The measures were analyzed with descriptive statistics and the preliminary results show a high level of acceptance and demand, from both patients and providers. Results also indicate successful initial implementation in the regional Department of Clinical Genetics and preliminary efficacy, as shown by significant clinically important improvement in patients' empowerment levels.

## What Is Known

Genetic counseling is well suited for telemedicine appointments; ie using telephone and video service delivery models. However, the video service delivery model is not frequently used. Implementation of complex healthcare innovations are known to be difficult to sustain.

## What Is New

Implementing telegenetic counseling in a certain Swedish context is feasible, from both a patient and provider perspective. These service delivery models are acceptable, in demand, and increase patients' empowerment after genetic counseling.

## Introduction

Before the COVID-19 pandemic, the Department of Clinical Genetics in Sweden's Southeast Healthcare region was examining an alternative service delivery model, specifically video appointments, to provide genetic counseling. The aims were to meet the growing demand for genetic investigations and counseling related to hereditary conditions, and to increase flexibility and equality of patient care (1, 2). Sustainable development goals of the United Nations were naturally included, such as *Good health and well-being; Reduced inequalities; Industry, Innovation and infrastructure* ((3): https://www.un.org/sustainabledevelopment/health/). However, prior to COVID-19 the projects progress was slow, in part due to barriers perceived by healthcare providers, such as lack of evidence and resources, both human and technological (1). As the COVID-19 pandemic surged in early 2020, traditional face-to-face appointments were no longer considered appropriate to deliver genetic counseling. The Department of Clinical Genetics, like many other clinics worldwide, rapidly transitioned from face-to-face to distance appointments, delivered by telephone or video, and from hereon referred to as Telegenetic counseling (TGC). Rarely appointments were postponed indefinitely (4–6). Telegenetic counseling is the focus of this study, and the Healthcare Region of Östergötland provided Visiba Care, a virtual clinic software solution, free of charge during the pandemic study period. Rapid implementation of telegenetic counseling became crucial to inhibit infectious spread and maintain adequate patient care, despite the on-going pandemic and previously stated barriers (1). Little was known regarding feasibility of telegenetic counseling from a patient perspective, and to fill the knowledge gaps this feasibility study was conducted. Four main aspects of feasibility were considered: acceptability, demand, implementation and efficacy of telegenetic counseling (7). Efficacy was measured using improvement in patient empowerment, as this is one of the main tennets of genetic counseling. Empowerment is defined as “*a set of beliefs that enable a person from a family affected by a genetic condition to feel that they have some control over and hope for the future”* (8). This study is, to our knowledge, the first to use findings on the empowerment effects for telegenetic counseling appointments as measured by the previously validated patient-related outcome measure (PROM) the Genetic Counseling Outcome Scale-24 (GCOS-24) (8, 9).

This study took place from May to December 2020 and the process started with healthcare professionals' discussion and evaluation meetings, followed by two PROMs previously validated in English: GCOS-24 (10) to measure empowerment, and Telehealth Usability Questionnaire (TUQ) (11) to measure usability. The preliminary findings and experiences of rapid implementation of telegenetic counseling are presented here.

## Background

Sweden offers all residents universal healthcare. The Department of Clinical Genetics in the Southeast healthcare region provides genetic services (including referrals for genetic investigation, testing and counseling) to approximately 1 million inhabitants, and is one of six regional genetics clinics in Sweden. At the time it was staffed by three consultant geneticists, seven resident physicians in clinical genetics, seven genetic counselors and several administrative staff. Specialities in genetics include oncology, cardiology, pediatric, prenatal and general genetics. The number of referrals for genetic counseling had increased by 47% between the year 2016 and 2020 [Clinical Genetics Dept.: Internal administration]

To meet increasing demands and reduce unequal access to genetics services, alternative service delivery models using e-health were being investigated. The concept e-health was defined as “*using information and communication technologies for health”* (12). One example of e-health is telegenetic counseling (TGC), which is already used by clinical genetic services worldwide (13–18). The definition of telegenetic counseling in this study is: *to provide genetic counseling via video or telephone, i.e., digitally and remotely*. Despite increasing interest in TGC, uptake has been consistently low in many countries, including in Sweden.

Historically telehealth has proven difficult to sustain due to barriers such as lack of integrated operation models (19) and barriers such as technological issues, lack of resources and evidence of effectiveness (1, 20), including effects on patient empowerment, experiences and preferences (21). However, a recent systematic review concluded that TGC is an acceptable alternative considering patients' psychosocial aspects, satisfaction and knowledge gain (18). However, the context for most included studies was in an English-speaking country, mostly at a satellite clinic to which the patient had to travel, and mainly for oncogenetic investigations.

TGC has not previously been studied in the Swedish context, from the chosen location of the patient, i.e., home. The Swedish society is considered one of the most digitalized in the world, ranked second on the Digital Economic and Society Index in 2020. This means that 95% of the Swedish population already use the internet daily for various reasons and that most households have fixed broadband and 4G coverage, making Sweden well equipped for telegenetic counseling (22).

This study has two distinct phases for implementation of TGC. First, a preparatory phase to investigate requirements and factors that could influence use of TGC among healthcare providers has previously been published (1).

For the second phase a feasibility approach was deemed appropriate, as there was already some research on telegenetic counseling, albeit in different contexts, such as in remote satellite clinics, in English only, without pandemic related requirements of physical distancing (13, 16, 18, 23–25). In general, feasibility studies can be useful to figure out important parameters to design a main study, like participants willingness to be recruited, and of clinicians to recruit; number of eligible patients to calculate power for larger studies; characteristics and suitability of outcome measures; response rates; and to identify necessary changes before larger studies (26). This approach is particularly useful when studying intervention proof-of-concept in a specific real-world setting, in order to determine appropriateness for larger efficacy studies (7).

We hypothesized that use of TGC in the Swedish healthcare setting was feasible from both providers' and patients' perspectives, and the aim was to investigate the preliminary feasibility of TGC after rapid implementation, by answering the following research questions:

**Acceptability:** Is TGC considered acceptible by healthcare providers (HCP) and patients?
**Demand:** Is there a demand for TGC from HCP and patients?
**Efficacy:** Is TGC efficacious in improving patient empowerment, and how does it compare to face-to-face genetic counseling?
**Implementation** What organizational changes are needed to facilitate implementation of TGC?

## Methods

### Participants, Recruitment and Setting

**Diagram 1:**
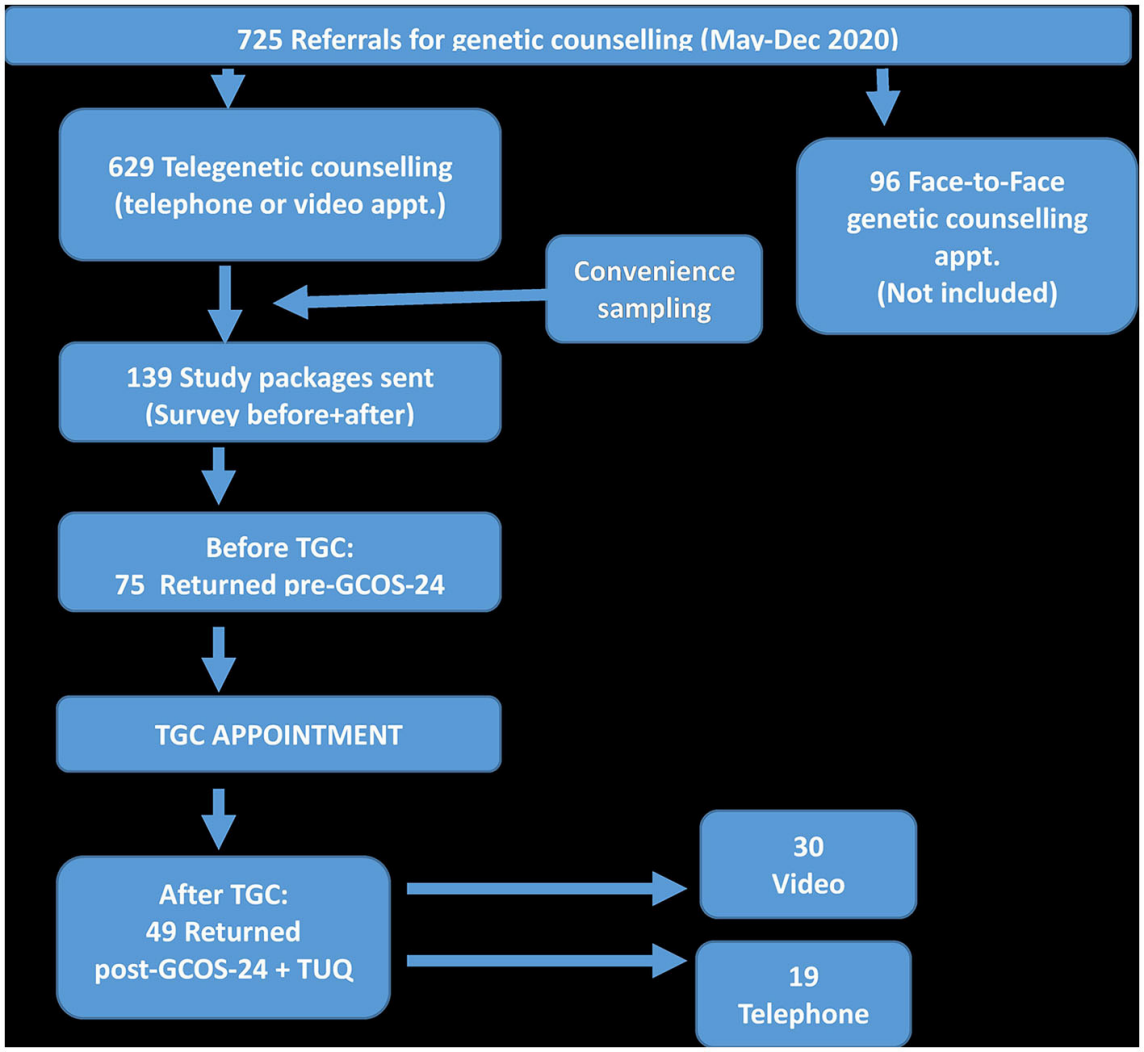
Flow chart showing the recruitment process of patient participants. Genetic counselling outcome scale-24 in Swedish (GCOS-24 Swe); Telegenetic counselling (TGC); Telehealth Usability Questionnaire in Swedish (TUQ).

Healthcare providers at the Department of Clinical Genetics triaged patients for eligibility to participate in the study, upon referral for genetic counseling. Using convenience sampling patients were allocated TGC-appointments in either the Telephone-group *or* the Video-group. Most appointments were telegenetic counseling, due to COVID-19 restrictions at this time. A few referrals were excluded during triage, mainly due to unusual complexity (i.e., syndromology), or very high emotional load, (i.e., Huntingtons Disease), or requiring an interpreter. The parents of patients under 18 years of age were offered to participate instead of the minor. Eligible patients, or their parents, were either directly sent the study information-package together with their appointment letter (Telephone-group), or called by study administrators to be informed about the study (Video-group) and sent the study information-package. The package included: *appointment instructions, participant information sheet, questionnaires [GCOS-24 before (pre-counseling), GCOS-24 after and TUQ (2 weeks post-counseling], a form for declining participation in the study and two paid postage return envelopes*. Participants were informed that participation in the study was voluntary, and would not affect their appointment in any way. The Video-group also had to fullfill the technical requirements, specifically: *access to a high-speed internet connection; a smart-device (telephone or tablet) or a computer; web camera, secure national personal identification application (Swedish Bank-ID), Google Chrome browser and E-mail address*. If these requirements were not fulfilled, or the patient declined the video appointment, the patient was automatically transfered to the Telephone-group instead. Consent to participate was given upon returning the pre-counseling questionnaire (GCOS-24). The paticipants were, if necessary, sent the two reminders four and 6 weeks after their appointments. Case preparation was done as usual by the healthcare provider, and was followed by the TGC-appointment. Participants could choose a preferred location for the appointment (i.e., either at work, at home or other location) that satisfied the instructions given: “*try to be in a quiet place, without disturbances; the appointment will take approximately one hour*.” HCPs and patients in the Video-group also completed a short evaluation *(provided by Visiba Care, described under Measures)* directly after each appointment. Diagram 1 shows the recruitment procedure of patient participants.

The participating HCPs comprised of six volunteering co-workers at the regional Department of Clinical Genetics in Linköping, Sweden. The group consisted of two consultant geneticists, one Resident Physician in Clinical genetics, two administrators and one EBMG-certified genetic counselor, whom is also the main author of this paper. The group was made up of one male and five females. Experience in clinical genetics and genetic counseling varied from 2–20 years. Three study-meetings were held with HCPs to discuss the acceptability, demand and implementation aspects. HCPs also discussed the in-house questionnaire (*described under Feasibility aspects below, and as* Supplemental Material).

### Feasibility Aspects

The study focused on the following feasibility aspects: *Acceptability, Demand, Efficacy and Implementation*, as described below. The measures used to capture these aspects are shown in Table 1.

**Table 1:** Measures used to capture different feasibility aspects.

**Feasibility** **Aspects**	**Study participant**	**Measure**	**TUQ**	**VCE**	**Org Info**	**DQ**
		**GCOS-24**				
Acceptability	Patient		x	x	x	
	Provider			x		x
Demand	Patient		x	x	x	
	Provider				x	x
Efficacy	Patient	x				
	Provider					
Implementation	Patient				x	
	Provider			x	x	x

*An overview of the different measures used and corresponding feasibility aspect. X indicates which participants answered. Genetic Counseling Outcome Scale-24 (GCOS-24), Telehealth Usability Questionnaire (TUQ), Visiba Care Evaluation (VCE), Organizational Information (Org Info), Descriptive Questionnaire (DQ)*.

#### Acceptability

This includes providers' and patients' reactions to telegenetic counseling: to what extent is it a suitable, satisfying and attractive alternative? (7). The measures used among providers were the in-house questionnaire (*that include 7 closed questions that captured provider experience, satisfaction and preferences; see* Supplemental Material *for details*) and the Visiba Care evaluation after each appointment *[“How did you experience the quality of the video appointement?” (*Answer 1; Very good −4; Very bad)]. To capture the patient perspective, the GCOS-24 (*See more details in the Measures section and in Swedish in* Supplemental Material), TUQ (*see more details in the Measures section and in Swedish in* Supplemental Material) and Visiba Care evaluation *[“How did you experience the quality of the video appointement?” (*Answer 1; Very good −4; Very bad)] from each appointment were used.

#### Demand

Demand can be designated by the documented use of TGC. The following questions are answered: *Does TGC fit with the organization? What is the perceived demand? What is the actual and intended use?* (7). To capture this aspect the number of referrals, offered, accepted and declined telegenetic counseling appointments were used. To capture the patients' perspective, the Visiba Care evaluation and TUQ questionnaire were used.

#### Efficacy

Change in empowerment is compared between pre-genetic counseling and post-genetic counseling, as a measure of the efficacy of TGC. The sample is small, providing low statistical power and therefore only preliminary efficacy can be measured (7). The GCOS-24 evaluations are used to capture the patients' change in empowerment when using TGC, and contrasted to face-to-face counseling on a group-level, by comparing with previously published, international GCOS-24 empowerment outcomes (9).

#### Implementation

This aspect involves the degree and status of execution, and identifying what resources are needed (7). To capture the providers' perspective, the Visiba Care evaluation and discussions from the pilot period are used, as well as identifying changes made in the organization to allow for implementation.

### Survey Measures

The Swedish GCOS-24 (The patient-reported outcome measure Genetic Counseling Outcome Scale-24, Table 1) was included to capture the effects of telegenetic counseling by measuring changes in patients' empowerment levels before genetic counseling compared to after genetic counseling. Patients completed participation in our study by answering GCOS-24 at two time-points: 1–4 weeks before the appointment and 1–4 weeks after the appointment. The Swedish GCOS-24 contains 24 questions regarding their experience of genetic counseling, and responses are given on a 7-point Lickert scale, ranging from 1; Strongly disagree to 7; Strongly agree (8, 10). Scoring is done by assigning each response 1–7 points and the final score can range from 24–168 points. (*see* Appendix A: *GCOS-24 Swedish*). Questions # 6, 14 and 21 have negative questions and are reversed before scoring. A mean increase more than 10.3 points, on a group level, indicates that minimal clinical important difference (MCID) has been achieved, thus providing clinical utility and interpretability to the scores (27). GCOS-24 is validated in English (8, 10), and has shown test–retest reliability (r = 0.86) and a medium-to-large effect size (Cohen's d = 0.70). It has been translated to different languages (Spanish, Danish, Portugease, Dutch) and used in different settings (Oncogenetics, Psychiatric genetics, General genetics) (9, 28–33). GCOS-24 was translated and cross-culturally adapted to the Swedish setting, following a modified protocol by Beaton (34): *Forward translation from English to Swedish was conducted by two independent translators. New versions were merged, and then back-translated by another translator. The back-translated version was consolidated with the original, and changes agreed upon by an expert group. Face-validity and semantic equivalence were assessed through cognitive interviews with patients or patient representatives (n* = *6 in our study). After reaching consensus in the expert group, the final version was used in this study*. (34, 35).

The Telehealth Usability Questionnaire (Table 1, TUQ) measures patients' perceptions of the usability of the telehealth technology (*telephone or video)*. The usability aspects include usefulness; ease of use; effectiveness; reliability; and satisfaction. Patients who completed the study answers the TUQ 1–4 weeks after their TGC-appointment. The TUQ contains 21 questions and responses are given on a 7-point Lickert scale. Scoring was done by assigning each answer 1–7 points, from 1; Strongly disagree to 7; Strongly agree (11). (*Question #14 regarding visiual experience only applied to video appointments, thus was excluded in comparative analysis*). The theoretical score ranged from 21–140 points. Validation has shown good to excellent reliability (Cronbach's Alpha coefficient of 0.81–0.93) in previous study (11). The questionnaire had been translated and cross-culturally adapted to Swedish following the same modified protocol by Beaton, as described above for GCOS-24 (34, 35).

Visiba Care evaluations (Table 1, VCE) were administered directly after each video appointment to both providers and patients. Providers were asked to score the quality of the video appointment from 1: lowest satisfaction to 4; highest satisfaction. Patients were asked to score four items relating to satisfaction, including inclination to recommend video appointments to others from 1: lowest satisfaction to 4; highest satisfaction.

The implementation aspect was captured by collecting organizational information from managerial staff. Information included: number of offered, accepted, and declined appointments, number of active providers using TGC, and necessary changes made in the organization to accommodate the rapid implementation of TGC (Table 1, Org Info).

A descriptive questionnaire was used to collect information on providers' experiences regarding acceptability, demand and implementation of telegenetic counseling. The questionnaire had seven closed questions collected only during the first month of the study. In addition, monthly meetings were held during the study period to discuss experiences using telegenetic counseling and satisfaction with the video appointments (Table 1, DQ and for details see Supplemental Material).

### Data Analysis

Statistical analyses were conducted with the software r in the interface software RStudio (36, 37). The GCOS-24, TUQ and VCE questionnaires were analyzed with descriptive statistical analysis. The DQ (descriptive questionnaire) with 7 closed questions, together with the provider meetings on HCP experiences are presented in summary under each relevant aspect in the Results section. Categorical data are presented as frequencies and percentages, and continuous data are described as means and standard deviations. Missing data were imputed with the multiple imputations by chained equations as implemented in the r package mice (38) with the default method to give 5 complete datasets. As recommended with this imputation method, all analyses were completed for all five datasets and the average results over the five datasets are presented. The questionnaires with missing data less than 5% were imputed based on previous answers, and those with more than 50% dropout were excluded. There was on average 1.9% missing data, which were treated as missing at random. The efficacy analyses (improvement in GCOS-24 and difference between video and telephone appointments in GCOS-24 or TUQ) were analyzed with Welch *t*-tests (which allows for unequal variances). Because this is a pilot study, the sample size has not been determined by statistical power. However, *post hoc* statistical power shows that the study has 95% statistical power for detecting the MCID of 10 points increase in GCOS-24. This is very good statistical power, but subgroup divisions will have lower power (like the difference between video and telephone appointments).

## Results

### Provider Participants

The provider study group was comprised of three clinical geneticists (2 senior with 10 and 20 years of experience in the field, and one junior with 2 years experience) and one senior genetic counselor with 12 years experience in the field (first author of this paper). The group included two experienced medical administrators to support the providers with administrative tasks related to the study and implementation process.

### Patient Participants

Of the 139 invited patients, seventy-five patients (75/139, 54% partial response rate) returned one of the three questionnaires (GCOS-24 before/GCOS-24 after/TUQ), and thus consented to participate. After their TGC appointments, fourty-nine patients (49/139, 35% complete response rate) returned all three questionnaires during the study period (see Table 2). The post—video appointment Visiba Care evaluation, administered to both patients and providers, had a response rate of 35%.

**Table 2:** Demographics of patient participants.

		**% (*n*)**
Total participants for all 3 questionnaires		100 (49)
Gender	Female	69 (34)
	Male	31 (15)
Age: Mean (range years)		47 yrs. (5–79)
Referral type	Hereditary cancer syndromes	61 (30)
	Other genetic inquiries	39 (19)
Appointment type	Telephone Video	39 (19) 61 (30)
Health care professional	Doctor (Dr)	57 (28)
(*Provider profession*)	Genetic counselor (GC)	33 (16)
	Both Dr and GC	10 (5)
Previous genetic counseling	Yes No	8 (4) 92 (45)

The patients were divided into different groups, based on inquiry type: Hereditary cancer syndromes, and Other genetic inquiries. The referrals for Hereditary cancer syndromes were dominated by: Hereditary breast and ovarian cancer; Lynch syndrome, and Familial pancreatic cancer and malignant melanoma. Other genetic inquiries included a range of genetic diagnosis such as; Spinal muscular atrophy; Polycystic kidney disease; Balanced translocation, and Maturity-onset diabetes in the young. For most participants this was their first experience with genetic counseling.

#### Feasibility Aspects for Telegenetic Counseling

The findings capture perspectives from both provider and patient.

### Acceptability

#### Patient Perspective

Out of 139 patients offered a TGC appointment, four patients (2.8%) declined. The stated reasons were “*lack of computer equipment*” or “*lack of technological competence*” [*communication from the project administrators*]. The Visiba Care evaluations showed a high level of mean patient satisfaction (*M* = 3.77 out of 4, *SD* = 0.47).

The TUQ measure included questions about patients' preference to use telegenetic counseling, even without an on-going pandemic. Thirty three patients (33/49 = 69%) answered that they would have preferred a video appointment in any case. Open-ended comments to the TUQ-questions showed that many patients desired this service delivery model in the future, indicating that telegenetic counseling is acceptable to patients, as illustrated by this quote; “*happy not to have to travel to the clinic*.” Patients seemed to consider genetic counseling appropriate to receive by telephone or video, since “*it was just a conversation*,” as stated by one participant.

#### Provider Perspective

During the study period the Visiba Care video system was used in 92 appointments (not all were invited to participate, due to convenience sampling) ranging between 13–23 appointments per HCP participant, whom used it at least once a week throughout the whole study period. The mean score in the Visiba Care evaluation on provider satisfaction was 3.42 (*SD* = 0.81) out of 4. Also responses to the in-house questionnaire (DQ) and in HCP study meetings indicated HCP satisfaction using telegenetic counseling, as illustrated by these quotes: “*provision of care amid a pandemic, despite physical distancing*,” “*no need to travel*,” “*increased accessibility*,” “*see patient and reactions to what is said*,” “*easy to use*” and “v*ery little technological difficulties*.” Differences between telephone and video appointments became apparent, as verbalized by one HCP: “*video is better than a phone call*.” Several negative aspects emerged regarding video appointments, but not regarding telephone appointments. For example, some providers did experience technical difficulties, such as poor internet connections, power-shortages, or a very small screen (when the patient was on a smartphone). Also mentioning increased preparatory work and tiredness afterwards caused some providers extra stress due to the novel work situation when using video appointments.

### Demand

#### Patient Perspective

A majority of patients (67%) preferred telegenetic counseling, compared to 30% that preferred a physical appointment, even without the COVID-19 pandemic, as found from TUQ. Most patients would recommend the use of video appointments to others, according to the mean score of 3.84 (*SD* = 0.37) out of 4 in the Visiba Care evaluation. Very few patients reported technical difficulties or disturbances, however, one patient indicated that there had been connection issues and difficulty hearing the provider in the video-appointment.

#### Provider Perspective

All of the six providers using the video platform during the study period (two administrators and four healthcare professionals) stated that they wished to continue with TGC appointments after the study. All of the staff at the clinic are now (April 2022) using video and telephone regularly. The time used for each video-appointment was reported, and varied (range 4 to 111 min), but the mean time (*M* = 32.8, *SD* = 17.4) was much shorter than compared to the 60 min time always allocated for face-to-face appointments.

### Efficacy

#### Patient Perspective

**Figure 1:**
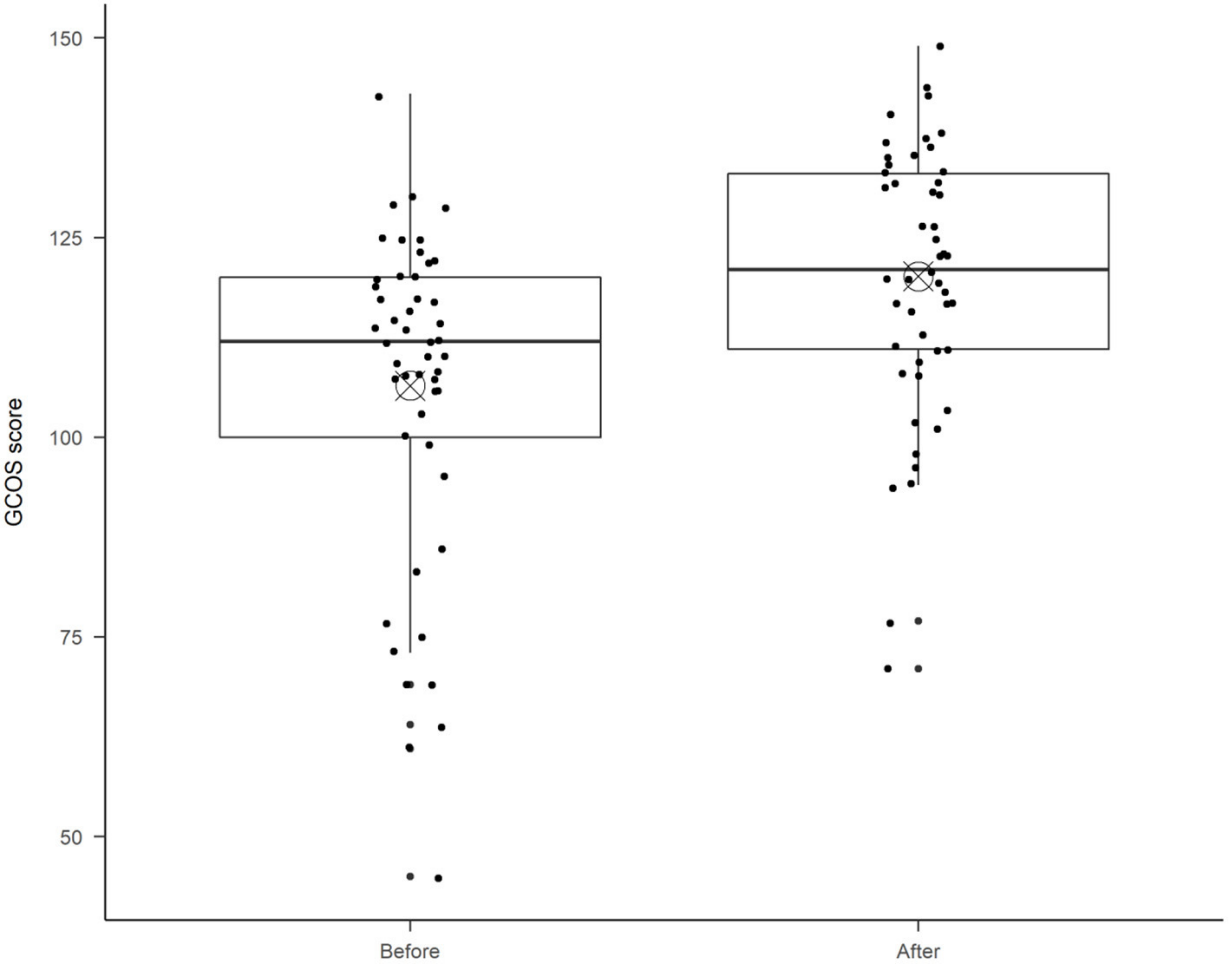
GCOS-24 scores before and after telegenetic counseling. The boxplot diagram shows the median (horizontal line), and the bull's eye indicates the mean in each boxplot. The ends of the box show the 25th and 75th percentile. Each dot represents one patient's total score.

A Welch *t*-test [*t*(48) = 6.54, *p* < 0.001] showed that empowerment, as measured with GCOS-24, was significantly higher after TGC (*M* = 120, *SD* = 16.9) than before TGC (*M* = 106, *SD* = 20.8), see Figure 1. The increase of 13.9 points (*SD* = 14.9) is above the identified MCID (27), which indicates that a clinically important difference in empowerment was achieved on a group level 2 weeks after telegenetic counseling.

**Figure 2:**
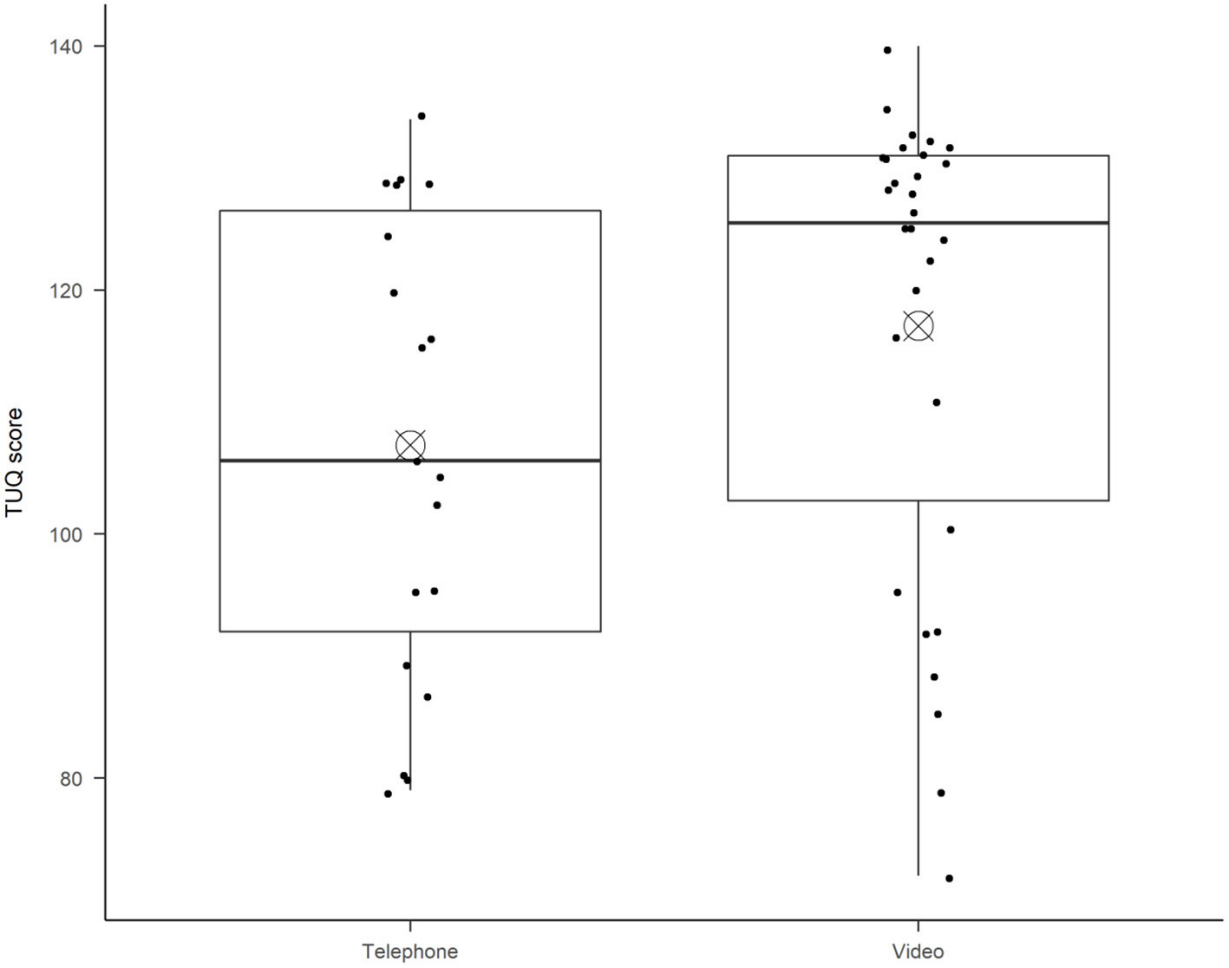
Shows usability ratings following the Telehealth usability questionnaire, divided by patients who had Telephone appointments or Video appointments. The boxplot diagram shows the median (horizontal line), and the bull's eye shows the mean in each boxplot. The ends of the box show the 25th and 75th percentile. Each dot represents one patient's total score.

This pilot study does not have enough statistical power for subgroup comparisons, but there was a non-significant tendency [Welch *t*-test, *t*(40.53) = 1.35, *p* = 0.19] the improvement in empowerment was higher for video appointments (*M* = 16.1, *SD* = 15.2) than for telephone appointments (*M* = 10.4, *SD* = 14.1), see Figure 2. There were also a non-significant tendency that the video appointment patients had lower empowerment before TGC (*M* = 104, *SD* = 21.9) as compared to the telephone appointment patients (*M* = 109, *SD* = 19.1). This should be followed up in later efficacy studies with statistical power for subgroup analyses.

**Figure 3:**
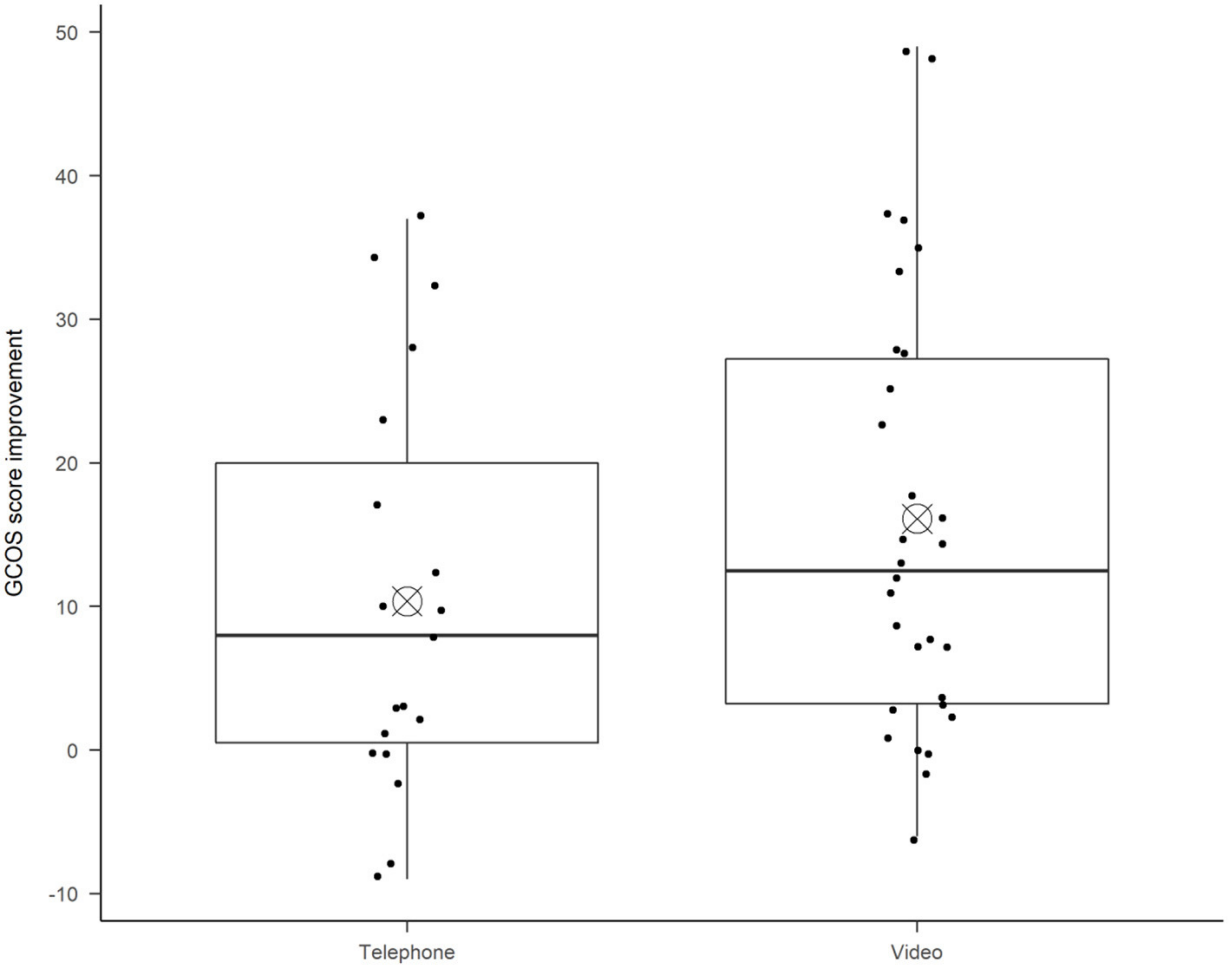
The diagram shows the difference in empowerment increase, divided by patients who had Telephone appointments or Video appointments. The boxplot diagram shows the median (horizontal line), and the bull's eye shows the mean in each boxplot. The ends of the box show the 25th and 75th percentile. Each dot represents one patient's total score improvement.

The mean rating from patients on usability of telehealth (TUQ) post-TGC was 113/140 (SD = 19.6), indicating that the patient group rated the systems as usable and effective. There was a non-significant tendency [Welch *t*-test, *t*(38.60) = 1.74, *p* = 0.09] that video appointment patients rated higher usability (*M* = 117, *SD* = 19.2) than telephone appointment patients (*M* = 107, *SD* = 19.1), see Figure 3.

### Implementation

#### Patient Perspective

Regarding necessary resources, i.e., technical equipment and competences, most patients fulfilled requirements (97.2%) and only 2.8% of patients declined appointments as they did not meet the necessary requirements or could not get technical support from family or friends.

#### Provider Perspective

The implementation process of TGC had in reality started before the COVID-19 pandemic in the Department of Clincial Genetics. In fact, the organization, management and staff were prepared and informed about the impending plans to implement TGC. When rapid implementation was forced by the pandemic, it was appreciated by both staff and management that genetic counseling could seamlessly continue for our patients, as found in the in-house questionnaire and workshop meetings with HCP participants. Multi-level organizational support (regional; clinical and clerical); staff involvement and management involvement in the project group (*one of the clinical geneticists is now the Head of Department*) during the whole process were necessary for rapid implementation. Other aspects affecting the implementation in our clinic were the regular HCP participant meetings and reports showing actual useage and progress, providing opportunities to discuss issues directly and make quick changes to resolve potential issues. For example, administrative changes and routines were made, then tested and evaluated and altered again, when necessary. To start administrators spent a lot of time calling patients to offer video appointments, as described in the Methods section, study protocol. After the study period (December 2020) this time-consuming routine has changed, and written offers of video and telephone appointments are only used. Other examples of implementation preparations included: Creating a check-list for Telegenetic counseling appointments; Providing a 1 h long, mandatory introduction-to-TGC-sessions to all staff; Improved administrative routines; Conducting concurrent evaluations (patients and staff); and Offering monthly, voluntary Q&A-sessions for staff with IT-professionals present. To date, all employees at the genetics clinic have started using the video system for telegenetic counseling, in addition to telephone appointments.

## Discussion

Our study shows that both patients and providers find using telegenetic counseling acceptable and that there is demand for it, which corresponds with conclusions from other studies (18). In addition, results from this study indicate successful implementation and preliminary efficacy (i.e., clinically improved empowerment) when using telegenetic counseling, which has to our knowledged not previously been reported.

The efficacy results on empowerment effects from telegenetic counseling are comparable to face-to-face genetic counseling (i.e., standard clinical care) on a group level. The mean empowerment increase was 13.9 which is comparable to reports in other international studies after face-to-face genetic counseling [such as 10.8 by (28), or the “*significant improvement in empowerment”* reported by other (39, 40)]. Another study on face-to-face psychiatric genetic counseling showed an empowerment increase of 18.5 (30), and post-genetic counseling scores (at 120.8) from a Danish face-to-face study (29) were also comparable to post-GC score (119.5) in the present study.

Satisfaction of TGC was overall high from both providers and patients, similar to findings from studies in various contexts (18, 23, 24, 41–43).

To our knowledge, this is the first study to measure effects on patient empowerment after telegenetic counseling. However, some would argue that similar effects on empowerment would be seen after *any* kind of supportive, digital encounter with a patient. Indeed, awareness of the *digital placebo effect* is important, as positive outcomes have been found from a patient just using a smart device for health purposes (44). Interestingly, patients in our video-group experienced non-significant higher levels of empowerment compared to patients in the telephone-group which differs from the findings in a systematic review where most studies did not show differences between telephone and video appointments (18). There can be different explanations to this finding, for example, selection bias during the triaging, as the two different groups had different base levels of empowerment before genetic counseling in our study (Telephone: 109 vs. Video: 104). Another reason could be that patients improved their understanding and sense of control through visual interactions such as visual aids, body language, facial expressions, or reading lips, a hypothesis supported by Voils et al. (45) that patients appreciate seeing the counselors body language. This explanation fits well with the Theory on therapeutic alliance; seeing the other person creates a rapport and relationship, which are both considered necessary for effective genetic counseling (46), smiliarly reported in at least one other mid-Covid study on TGC (5). The therapeutic alliance requires a positive, safe relationship between patient and provider, which is made easier by seeing one another. To understand this phenomenon larger studies are necessary to compare empowerment outcomes between telephone, video and face-to-face genetic counseling, and to assess the therapeutic alliances using for example the working alliance inventory (46).

Results indicate a continuing demand for telegenetic counseling from both patients and providers. The flexibility, cost- and time-saving benefits mentioned by participants correlate well with previous reports on telegenetic benefits (14, 18, 24, 47). Telegenetic services are seen as a way to meet the growing demands for genetic counseling, and now also reducing viral spread (4, 5). However, telegenetic counseling, does not fit all patients, providers or inquiries. Aiming to reduce inequalities and improve good health and well-being, TGC should be considered one of several alternative service delivery models to provide increased flexibility and equality to patients, regardless of where they live or how mobile or tech-savvy they are UN: United Nations (3).

Telegenetic counseling can be considered successfully implemented in this study, potentially due to the motivation, needs and involvement of involved healthcare providers, which have been identified as important factors for a successful implementation and acceptability of telehealth (48, 49). Interestingly, acceptance of telegenetic counseling appears to have increased over the past few years, when compared with findings in our previous study (1). Those participating healthcare professionals showed a more hesitant stance, and a natural explanation could for example be the change in context due to COVID-19, thus adding a whole new benefit to using telegenetic counseling not mentioned previously. As described by West and Michies' (50) summay on the COM-B model of behavior: “*a particular behavior will occur only when the person concerned has the capability and opportunity to engage in the behavior and is more motivated to enact that behavior than any other behaviors*.” As the pandemic arose the physical and social opportunities increased, which according to COM-B influences the persons' motivation to change their behavior. Once the person has learned a new, complex skill such as using TGC technologies, and this skill is practiced, the persons capability will improve and further increase their motivation to engage in the behavior (50). This is most likely also the reason behind the surge in publications on telegenetics since the beginning of the COVID-19 pandemic [(4, 6, 51–53), *and many more*].

Several favorable implemention circumstances were identified during this study, and fit well with a concept of *Telehealth readiness* (54). The circumstances that improved our outcome were among others: *Prior preparation*—in reality, the process of change is slow and requires a long period of mental preparation. *Support*—a collective effort that requires all different stakeholders to get involved and together create an environment where ideas and innovations can thrive. *External circumstances*—when the alternatives (*in our case: no genetic counseling*) are worse than the proposed intervention (*in our case: telegenetic counseling*). *Right timing*—when aspects beyond control occur to create favorable circumstances for the intervention, such as COVID-19 pandemic. These circumstances can be compared to earlier studies on telegenetic counseling, by for example (20), when instead several circumstances were unfavorable. For example 37% of their patients reported technical issues, and 1/3 of patients did not meet technical requirements (24). Accurately though, the authors hypothesized that these factors were likely to change in the near future.

### Study Limitations

Limitations in our study include the lack of a control group, i.e., face-to-face genetic counseling, or no genetic counseling. However, at the time of the study, it was not recommended to provide face-to-face counseling, due to COVID-19. Hopefully, in the near future, it will be possible to establish if similar empowerment improvements are achieved in a face-to-face group in the Swedish context. Another limitation is the small sample size of patients and providers, which does not render enough statistical power to draw far-reaching conclusions, but rather gives only indications, that are applicable in the very specific Swedish clinic, mid-COVID context. Furthermore, sampling bias may have occurred during triaging and by using the convenience sampling method. This could have been controlled by randomization of participants to different groups. Another aspect to try to improve in future studies is the response rate on the patient-reported questionnaires. Various patient-related reasons could explain the low response rate in our study, like canceling appointments, or forgetting to fill in, or not wanting to fill in the questionnaires. In light of stated limitations, the findings of this study can be considered preliminary evidence, and additional studies are needed (18).

## Conclusions

This study shows that telegenetic counseling is feasible, according to both providers and patients, specifically regarding the acceptability, demand, efficacy and implementation aspects. Results show that amidst the COVID-19 pandemic there was an increased demand for telegenetic counseling, and that implementation in the Department of Clinical Genetics in the Southeast region of Sweden was successful. The improved levels of patient empowerment in our study are comparable to face-to-face genetic counseling and are above the reported MCID. Despite the initial positive findings regarding feasibility in this study, it is important for individuals to be aware of potential hurdles when implementing new models of care, such as non-adoption, abandonment, and scale-up. The use of a guiding framework, such as the NASSS (non-adoption, abandonment, scale-up, spread, and sustainability) to identify, understand and address the long term challenges of implementing complex healthcare technologies could be helpful to use (55). This may inform future studies in the field and have implications for clinical practice when choosing service delivery models for genetic counseling, and providing more flexibility, whilst maintaining efficacy and satisfaction, despite an ongoing pandemic.

## Data Availability Statement

The raw data supporting the conclusions of this article will be made available by the authors, without undue reservation.

## Ethics Statement

The studies involving human participants were reviewed and approved by National Ethical Research Approval Authority in Sweden (Etikprövningsmyndigheten) Dnr: 2019-01051 on 2019-03-08. The patients/participants provided their written informed consent to participate in this study.

## Author Contributions

RP, PJ, and CG: conceptualization–ideas, formulation or evolution of overarching research goals and aims, writing—original draft—preparation, creation and presentation of the published work, and specifically writing the initial draft (including substantive translation). RP: project administration–management and coordination responsibility for the research activity planning and execution. RP, PJ, CG, HD, and MN: writing—review and editing—preparation, creation and presentation of the published work by those from the original research group, specifically critical review, commentary or revision—including pre-or post-publication stages. RP and HD: formal analysis—application of statistical, mathematical, computational, or other formal techniques to analyze or synthesize study data, visualization–preparation, creation and presentation of the published work, and specifically visualization/data presentation. All of the authors gave final approval of this version to be published and agree to be accountable for all aspects of the work in ensuring that questions related to the accuracy or integrity of any part of the work are appropriately investigated and resolved. All authors contributed to the article and approved the submitted version.

## Conflict of Interest

The authors declare that the research was conducted in the absence of any commercial or financial relationships that could be construed as a potential conflict of interest.

## Publisher's Note

All claims expressed in this article are solely those of the authors and do not necessarily represent those of their affiliated organizations, or those of the publisher, the editors and the reviewers. Any product that may be evaluated in this article, or claim that may be made by its manufacturer, is not guaranteed or endorsed by the publisher.
