# Supplementary material for: Rapid Implementation of Telegenetic Counseling in the COVID-19 and Swedish Healthcare Context: A Feasibility Study
Source: Front Health Serv. 2022 Jun 23;2:848512. doi: 10.3389/frhs.2022.848512 (PMC10012799; doi:10.3389/frhs.2022.848512)
Supplement: Supplementary file 1 [file Data_Sheet_1.PDF]

HCP questionnaire TGC Date ______________________________

Survey for healthcare professionals

Evaluation of distance visits for genetic counselling

We kindly ask that you complete and submit this questionnaire after you had a distance visit with a patient for genetic counselling. This information is collected so that the clinic can better understand how the TGC affects HCP who use it. By distance visit we mean genetic counselling conversations with the patient via telephone or video.

1. What type of visit did you have?

☐ Telephone (ie audio only)

☐ Video (ie audio and video)

☐ Physical (ie meeting at the clinic)

2. What type of visit do you usually prefer?

☐ Telephone (ie audio only)

☐ Video (ie audio and video)

☐ Physical (ie meeting at the clinic)

Was it your choice to have genetic counselling at a distance?

☐ Yes

☐ No

HCP questionnaire TGC Date ______________________

4. How many people attended during your distance visit?

☐ 1

☐ 2-3

☐ 4-5

☐ > 5

5. Had you met the same patient before?

☐ Yes

☐ No

☐ Do not remember

6. How did you experience the distance visit?

☐ Better than expected

☐ As expected

☐ Worse than expected

7. Other comments on how the visit was experienced (eg technical problems, other problems, something particularly good):
