# Supplementary material for: Rapid Implementation of Telegenetic Counseling in the COVID-19 and Swedish Healthcare Context: A Feasibility Study
Source: Front Health Serv. 2022 Jun 23;2:848512. doi: 10.3389/frhs.2022.848512 (PMC10012799; doi:10.3389/frhs.2022.848512)
Supplement: Supplementary file 2 [file Data_Sheet_2.PDF]

EFTER BESÖK (Endast TUQ för distansbesök GV) KOD-nr ______________________

Utvärdering efter distansbesök för genetisk vägledning

Vi ber att du fyller i och skickar in denna enkät inom 14 dagar EFTER ditt distansbesök. Använd svarskuvertet. Med distansbesök menar vi genetisk vägledningssamtal med personal från Klinisk genetik via telefon eller video. Kontakakta forskare Rebecka Pestoff på mejlen för frågor om enkäterna: rebecka.pestoff@regionostergotland.se.

Persondata skyddas genom att vara kodade.

Instruktioner:

1. Vänligen läs igenom deltagarinformationen (se separat blad)
2. Fyll i information om dig själv och ditt besök nedan
3. Besvara enkäten om din upplevelse av distansbesöket för genetisk vägledning

Om mitt besök:

1. Vilken typ av besök hade du?
   - ☐ Telefon (dvs endast ljud)
   - ☐ Video (dvs ljud och bild)
   - ☐ Fysiskt (dvs träffas för besöket)

2. Vilken yrkesroll träffade du vid ditt distansbesök?
   - ☐ Läkare
   - ☐ Genetisk vägledare
   - ☐ Båda
   - ☐ Vet ej

3. Hade du träffat samma vårdgivare tidigare?
   - ☐ Ja
   - ☐ Nej
   - ☐ Vet ej

4. Var det ditt val att ha genetisk vägledning på distans?
   - ☐ Ja
   - ☐ Nej

6. Skulle du välja besök för genetisk vägledning via distans igen i framtiden?
   - ☐ Ja
   - ☐ Nej

7. Om ja – Av vilken/vilka anledning/-ar skulle du välja telefon/videomottagning?

----

8. Övriga kommentarer om distansbesök:

➔ Fortsätt till **enkäten på nästa sida**

EFTER BESÖK (Endast TUQ för distansbesök GV) KOD-nr ____________________

Frågor om hur nöjd Du är med Ditt distansbesök för genetisk vägledning

Nedan finner du ett antal påståenden. Sätt ett kryss i den ruta där du mest instämmer med påståendet. Vänligen besvara alla frågor så gott du kan. För frågor som inte gäller dig välj alternativet: ”varken instämmer eller inte”.

**Svar**		Instämmer inte alls	Instämmer mycket lite	Instämmer lite	Varken instämmer eller inte	Instämmer	Instämmer mycket	Instämmer helt och hållet
1	Distansbesök gör hälso- och sjukvården mer tillgänglig för mig	◯	◯	◯	◯	◯	◯	◯
2	Distansbesök besparar mig restid vid hälso- och sjukvårdsbesök	◯	◯	◯	◯	◯	◯	◯
3	Distansbesök tillgodoser mina behov av genetisk vägledning	◯	◯	◯	◯	◯	◯	◯
4	Systemet för distansbesök var enkelt att lära mig använda	◯	◯	◯	◯	◯	◯	◯
5	Systemet för distansbesök var enkelt att använda	◯	◯	◯	◯	◯	◯	◯
6	Jag tror att jag snabbt skulle kunna bli duktig på att använda systemet för distansbesök	◯	◯	◯	◯	◯	◯	◯
7	Jag gillar att använda systemet för distansbesök	◯	◯	◯	◯	◯	◯	◯
8	Det är trevligt att använda systemet för distansbesök	◯	◯	◯	◯	◯	◯	◯
9	Systemet för distansbesök är enkelt och lätt att förstå	◯	◯	◯	◯	◯	◯	◯
10	Systemet distansbesök klarar allt som jag vill att det ska klara av	◯	◯	◯	◯	◯	◯	◯
11	Det var lätt att prata med vårdpersonal genom systemet för distansbesök	◯	◯	◯	◯	◯	◯	◯
12	Jag kunde höra vårdpersonalen tydligt vid användning av systemet för distansbesök	◯	◯	◯	◯	◯	◯	◯

EFTER BESÖK (Endast TUQ för distansbesök GV) KOD-nr ______________________

Svar		Instämmer inte alls	Instämmer mycket lite	Instämmer lite	Varken instämmer eller inte	Instämmer	Instämmer mycket	Instämmer helt och hållet
13	Jag kände att jag kunde uttrycka mig så som jag ville	◯	◯	◯	◯	◯	◯	◯
14	Med systemet för distansbesök kunde jag se vårdpersonal lika bra som om vi träffats på riktigt (OBS! gäller endast vid videobesök)	◯	◯	◯	◯	◯	◯	◯
15	Jag tycker att distansbesök är detsamma som att träffas på riktigt	◯	◯	◯	◯	◯	◯	◯
16	När jag gjorde ett misstag i användningen av systemet för distansbesök kunde jag lätt och snabbt fixa det	◯	◯	◯	◯	◯	◯	◯
17	Systemet gav felmeddelanden som tydligt talade om hur felet skulle fixas	◯	◯	◯	◯	◯	◯	◯
18	Jag känner mig bekväm med att använda systemet för att kommunicera med vårdpersonalen	◯	◯	◯	◯	◯	◯	◯
19	Distansbesök är ett godtagbart sätt att få genetisk vägledning	◯	◯	◯	◯	◯	◯	◯
20	Jag skulle använda distansbesök igen vid behov	◯	◯	◯	◯	◯	◯	◯
21	Överlag är jag nöjd med detta system för distansbesök för genetisk vägledning	◯	◯	◯	◯	◯	◯	◯

Tack för din medverkan!
