# Supplementary material for: Rapid Implementation of Telegenetic Counseling in the COVID-19 and Swedish Healthcare Context: A Feasibility Study
Source: Front Health Serv. 2022 Jun 23;2:848512. doi: 10.3389/frhs.2022.848512 (PMC10012799; doi:10.3389/frhs.2022.848512)
Supplement: Supplementary file 3 [file Data_Sheet_3.PDF]

Frågor om genetisk vägledning och ärftlig diagnos

Dina svar är viktiga för att utveckla och ytterligare förbättra omhändertagandet av våra patienter.
Alla frågor gäller det medicinska tillstånd, eller eventuellt ärftlig diagnos, som du eller någon annan i din släkt har. Stort tack på förhand för att du hjälper oss med detta. Hör av dig till forskarna om du har några frågor (doktorand Rebecka Pestoff: 010-103 1977 eller Överläkare Cecilia Gunnarsson: 010-103 6551).

All information kommer avidentifieras.

Instruktioner:

1. Vänligen läs igenom deltagarinformationen (se separat blad)
2. Fyll i information om dig själv nedan på denna sida
3. Besvara enkäten om din upplevelse av den ärftliga diagnosen i släkten och av den genetiska vägledningen

Om mig:

Kön ☐Kvinna ☐Man ☐Annan

Ålder ________________ år

Vilket medicinskt tillstånd söker du Klinisk genetik för?

☐________________________

☐Vet ej

Har du själv detta tillstånd? ☐Ja ☐Nej ☐Vet ej

Har någon i din släkt detta tillstånd? ☐Ja ☐Nej ☐Vet ej

Har du tidigare fått genetisk vägledning? ☐Ja ☐Nej ☐Vet ej

Har du barn? ☐Ja ☐Nej ☐Vill ej svara

➔ **Fortsätt till enkäten på nästa sida**

Linköpings universitet
Region Östergötland
Centrum för Sällsynta Diagnoser Sydöst
Region Jönköpings län
Region Kalmar län
Region Östergötland

LINKÖPINGS UNIVERSITET

Region Östergötland

Frågor om genetisk vägledning och ärftlig diagnos

Nedan finner du ett antal påståenden. Sätt ett kryss i den ring i vilken utsträckning du instämmer med påståendet. Vänligen besvara alla påståenden så gott du kan. För påståenden som inte gäller dig välj alternativet: "varken instämmer eller inte". Alla påståenden gäller det medicinska tillståndet (= eventuellt ärftliga diagnosen) som du eller någon annan i din släkt har.

**Svar**		Instämmer inte alls	Instämmer mycket lite	Instämmer lite	Varken instämmer eller inte	Instämmer	Instämmer mycket	Instämmer helt och hållet
1	Jag förstår varför jag besöker den genetiska mottagningen	◯	◯	◯	◯	◯	◯	◯
2	Jag kan förklara vad det medicinska tillståndet innebär för de familjemedlemmar som kan behöva veta	◯	◯	◯	◯	◯	◯	◯
3	Jag är medveten om vilken påverkan det medicinska tillståndet kan ha på mitt/mina (eventuellt framtida) barn	◯	◯	◯	◯	◯	◯	◯
4	Jag blir bekymrad när jag tänker på det medicinska tillståndet i min familj	◯	◯	◯	◯	◯	◯	◯
5	Jag vet vart jag ska vända mig för att få det medicinska stöd som jag och/eller min familj behöver (tex förbyggande åtgärder, mediciner, behandling, kontroller)	◯	◯	◯	◯	◯	◯	◯
6	Jag tycker att det medicinska tillståndet i min familj har lett till något positivt	◯	◯	◯	◯	◯	◯	◯
7	Jag upplever att jag har kontroll över hur det medicinska tillståndet påverkar min familj	◯	◯	◯	◯	◯	◯	◯
8	Jag känner mig positiv inför framtiden	◯	◯	◯	◯	◯	◯	◯
9	Jag känner att jag kan hantera att ha det medicinska tillståndet i min familj	◯	◯	◯	◯	◯	◯	◯
10	Jag vet vad jag har för nytta av de alternativ som finns tillgängliga för mig (tex genetisk testning, delta i kontrollprogram, fosterdiagnostik)	◯	◯	◯	◯	◯	◯	◯
11	Att ha det medicinska tillståndet i min familj gör mig orolig	◯	◯	◯	◯	◯	◯	◯
12	Jag vet hur det medicinska tillståndet kan påverka mina övriga släktingar på något sätt (tex syskon, farbröder/morbröder, fastrar/mostrar, kusiner)	◯	◯	◯	◯	◯	◯	◯

Nedan finner du ett antal påståenden. Sätt ett kryss i den ruta där du mest instämmer med påståendet. Vänligen besvara alla frågor så gott du kan. För frågor som inte gäller dig välj alternativet: ”varken instämmer eller inte”. Alla frågor gäller det medicinska tillståndet (= eventuellt ärftliga diagnosen) som du eller någon annan i din släkt har.

**Svar**		**Instämmer inte alls**	**Instämmer mycket lite**	**Instämmer lite**	**Varken instämmer eller inte**	**Instämmer**	**Instämmer mycket**	**Instämmer helt och hållet**
**13**	Mina beslut rörande det medicinska tillståndet kan påverka framtiden för mitt/mina (eventuellt framtida) barn	○	○	○	○	○	○	○
**14**	Jag förstår varför jag har en remiss till genetiska mottagningen	○	○	○	○	○	○	○
**15**	Jag vet hur jag kan få övrigt stöd jag och/eller min familj kan behöva (t.ex. från kurator, Försäkringskassan, kommunen, ekonomiskt stöd, socialt stöd)	○	○	○	○	○	○	○
**16**	Jag kan förklara vad det medicinska tillståndet innebär för personer utanför familjen som kan behöva veta (t ex vänner, skola, socialtjänst, arbetsplats, habilitering)	○	○	○	○	○	○	○
**17**	Jag vet vad jag kan göra för att förändra hur det medicinska tillståndet påverkar mig eller mitt/mina (eventuellt framtida) barn	○	○	○	○	○	○	○
**18**	Jag vet vilka i familjen som riskerar att utveckla det medicinska tillståndet	○	○	○	○	○	○	○
**19**	Jag tror att mitt/mina (eventuellt framtida) barn kan få ett så gott liv som möjligt	○	○	○	○	○	○	○
**20**	Jag kan planera för framtiden	○	○	○	○	○	○	○
**21**	Jag har dåligt samvete för att jag eventuellt kan föra det medicinska tillståndet vidare till mitt/mina (eventuellt framtida) barn	○	○	○	○	○	○	○
**22**	Jag känner mig maktlös att påverka något angående det medicinska tillståndet i min familj	○	○	○	○	○	○	○
**23**	Jag förstår varför jag har kontakt med genetiska mottagningen	○	○	○	○	○	○	○
**24**	Med kunskaper om det medicinska tillståndet kan jag fatta beslut som kan påverka framtiden för mitt/mina (eventuellt framtida) barn	○	○	○	○	○	○	○

Tack för din medverkan!
